# Evaluating Hospital Admission Data as Indicators of COVID-19 Severity: A National Assessment in Qatar

**DOI:** 10.1093/ofid/ofaf098

**Published:** 2025-02-17

**Authors:** Layan Sukik, Hiam Chemaitelly, Houssein H Ayoub, Peter Coyle, Patrick Tang, Mohammad R Hasan, Hadi M Yassine, Asmaa A Al Thani, Zaina Al-Kanaani, Einas Al-Kuwari, Andrew Jeremijenko, Anvar Hassan Kaleeckal, Ali Nizar Latif, Riyazuddin Mohammad Shaik, Hanan F Abdul-Rahim, Gheyath K Nasrallah, Mohamed Ghaith Al-Kuwari, Adeel A Butt, Hamad Eid Al-Romaihi, Mohamed H Al-Thani, Abdullatif Al-Khal, Roberto Bertollini, Laith J Abu-Raddad

**Affiliations:** Infectious Disease Epidemiology Group, Weill Cornell Medicine-Qatar, Cornell University, Doha, Qatar; Infectious Disease Epidemiology Group, Weill Cornell Medicine-Qatar, Cornell University, Doha, Qatar; Department of Population Health Sciences, Weill Cornell Medicine, Cornell University, New York, New York, USA; Mathematics Program, Department of Mathematics and Statistics, College of Arts and Sciences, Qatar University, Doha, Qatar; Department of Biomedical Science, College of Health Sciences, QU Health, Qatar University, Doha, Qatar; Hamad Medical Corporation, Doha, Qatar; Wellcome-Wolfson Institute for Experimental Medicine, Queens University, Belfast, UK; Department of Pathology, Sidra Medicine, Doha, Qatar; Department of Pathology and Molecular Medicine, McMaster University, Hamilton, Canada; Department of Biomedical Science, College of Health Sciences, QU Health, Qatar University, Doha, Qatar; Biomedical Research Center, QU Health, Qatar University, Doha, Qatar; Department of Biomedical Science, College of Health Sciences, QU Health, Qatar University, Doha, Qatar; Biomedical Research Center, QU Health, Qatar University, Doha, Qatar; Hamad Medical Corporation, Doha, Qatar; Hamad Medical Corporation, Doha, Qatar; Hamad Medical Corporation, Doha, Qatar; Hamad Medical Corporation, Doha, Qatar; Hamad Medical Corporation, Doha, Qatar; Hamad Medical Corporation, Doha, Qatar; Department of Public Health, College of Health Sciences, QU Health, Qatar University, Doha, Qatar; Department of Biomedical Science, College of Health Sciences, QU Health, Qatar University, Doha, Qatar; Biomedical Research Center, QU Health, Qatar University, Doha, Qatar; Primary Health Care Corporation, Doha, Qatar; Department of Population Health Sciences, Weill Cornell Medicine, Cornell University, New York, New York, USA; Hamad Medical Corporation, Doha, Qatar; Department of Medicine, Weill Cornell Medicine, Cornell University, New York, New York, USA; Ministry of Public Health, Doha, Qatar; Ministry of Public Health, Doha, Qatar; Hamad Medical Corporation, Doha, Qatar; Ministry of Public Health, Doha, Qatar; Infectious Disease Epidemiology Group, Weill Cornell Medicine-Qatar, Cornell University, Doha, Qatar; Department of Population Health Sciences, Weill Cornell Medicine, Cornell University, New York, New York, USA; Department of Public Health, College of Health Sciences, QU Health, Qatar University, Doha, Qatar; College of Health and Life Sciences, Hamad bin Khalifa University, Doha, Qatar

**Keywords:** Cohen's kappa statistic, COVID-19, hospitalization, sensitivity, severity

## Abstract

**Background:**

Accurately assessing SARS-CoV-2 infection severity is essential for understanding the health impact of the infection and evaluating the effectiveness of interventions. This study investigated whether SARS-CoV-2–associated hospitalizations can reliably measure true COVID-19 severity.

**Methods:**

The diagnostic accuracy of SARS-CoV-2–associated acute care and ICU hospitalizations as indicators of infection severity was assessed in Qatar from 6 September 2021 to 13 May 2024. WHO criteria for severe, critical, and fatal COVID-19 served as the reference standard. Two indicators were assessed: (1) any SARS-CoV-2–associated hospitalization in acute care or ICU beds and (2) ICU-only hospitalizations.

**Results:**

A total of 644 176 SARS-CoV-2 infections were analyzed. The percent agreement between any SARS-CoV-2–associated hospitalization (acute care or ICU) and WHO criteria was 98.7% (95% confidence interval (CI), 98.6–98.7); however, Cohen's kappa was only 0.17 (95% CI, 0.16–0.18), indicating poor agreement. Sensitivity, specificity, PPV, and negative predictive value were 100% (95% CI, 99.6–100), 98.7% (95% CI, 98.6–98.7), 9.7% (95% CI, 9.1–10.3), and 100% (95% CI, 100–100), respectively. For SARS-CoV-2–associated ICU-only hospitalizations, the percent agreement was 99.8% (95% CI, 99.8–99.9), with a kappa of 0.47 (95% CI, 0.44–0.50), indicating fair-to-good agreement. Sensitivity, specificity, PPV, and negative predictive value were 46.6% (95% CI, 43.4–49.9), 99.9% (95% CI, 99.9–99.9), 47.9% (95% CI, 44.6–51.2), and 99.9% (95% CI, 99.9–99.9), respectively.

**Conclusions:**

Generic hospital admissions are unreliable indicators of COVID-19 severity, whereas ICU admissions are somewhat more accurate. The findings demonstrate the importance of applying specific, robust criteria—such as WHO criteria—to reduce bias in epidemiological and vaccine effectiveness studies.

## Teaser

SARS-CoV-2–associated hospitalizations are unreliable for assessing COVID-19 severity, with a positive predictive value (PPV) of only 9.7%. Intensive care unit (ICU) hospitalizations have a higher PPV of 47.9% but low sensitivity at 46.6%. World Health Organization (WHO) severity criteria minimize bias in epidemiological assessments.

SARS-CoV-2 infection presents with a spectrum of symptoms, including the potentially fatal COVID-19 [1]. Accurate assessment of SARS-CoV-2 infection severity is critical for understanding the impact of the virus and its variants on public health [2–4], guiding the implementation of public health measures [5, 6], informing the development of vaccines and treatments [7–9], and evaluating the effectiveness of vaccines, treatments, and other interventions [10, 11]. A reliable tool for assessing infection disease severity is therefore essential. In response, the World Health Organization (WHO) developed standardized classification criteria to define COVID-19 severity [12], criticality [12], and fatality [13].

Hospitalization associated with SARS-CoV-2 infection is often used as a proxy for COVID-19 severity; however, this approach may not reliably or accurately reflect the true severity of the infection [14–16]. SARS-CoV-2 infection is sometimes diagnosed incidentally during hospitalization for unrelated conditions [14–16]. Additionally, hospitalization may occur for reasons unrelated to COVID-19 severity, including isolation protocols (as applied early in the COVID-19 pandemic), exacerbation of preexisting conditions, precautionary measures for individuals with underlying health issues or advanced age, or an inability to manage symptoms at home [2, 14–17].

Qatar appears to be the only country that has consistently applied the standardized WHO criteria to assess COVID-19 severity at a national level from the onset of the pandemic to the present [2, 16, 18]. This presents an opportunity to evaluate the extent to which hospitalizations associated with SARS-CoV-2 infection can serve as a reliable proxy for true COVID-19 severity. Therefore, this study was conducted to assess the diagnostic accuracy of acute care and intensive care unit (ICU) hospitalizations associated with SARS-CoV-2 infection as indicators of infection severity, compared to the reference standard of the WHO criteria for COVID-19 severity [12, 13].

## METHODS

### Study Population and Data Sources

The study was conducted on Qatar's resident population from 6 September 2021 to 13 May 2024. Data on COVID-19 laboratory testing, hospitalizations, and mortality were obtained from Qatar's national integrated digital health information platform (Supplementary Section S1). This platform has recorded all SARS-CoV-2–related data at the national level since the onset of the pandemic, including COVID-19 vaccinations, WHO criteria severity assessments of hospitalizations associated with SARS-CoV-2 infection (Supplementary Section S2), and results from both polymerase chain reaction (PCR) tests and medically supervised rapid antigen (RA) tests, regardless of location or healthcare facility (Supplementary Section S3). These databases also include information on coexisting conditions (Supplementary Section S4).

Qatar's SARS-CoV-2 testing program was extensive, with the majority of infections detected through routine testing rather than symptomatic presentation [18, 19]. Additional details on Qatar's population and COVID-19 databases have been previously reported [17–22].

### Acute Care and ICU Hospitalizations Associated With SARS-CoV-2 Infections

According to national COVID-19 guidelines, an acute care hospital admission associated with SARS-CoV-2 infection was defined as an admission to an acute care bed for an individual with an “active” SARS-CoV-2 infection, regardless of the individual's clinical condition or any specific hospitalization encounter codes [16]. Initially, the duration of active infection was set at 21 days from the date of a positive SARS-CoV-2 test. However, this period was revised to 14 days on 1 July 2020 and further reduced to 5 days on 17 October 2022, in alignment with national policy updates [16].

An ICU admission associated with SARS-CoV-2 infection was defined as an admission to an ICU bed for an individual with an active SARS-CoV-2 infection, where the ICU admission clinical team also determined that the condition could be related to COVID-19 [16]. In accordance with the national protocol, if an ICU admission for an individual with an active SARS-CoV-2 infection was deemed unrelated to COVID-19, the case was reclassified as an acute care admission associated with SARS-CoV-2 infection [16].

In this study, to account for the changes in the definition of active SARS-CoV-2 infection over time, a modified definition was applied for acute care and ICU admissions associated with SARS-CoV-2 infection. The duration of active infection was standardized to 14 days throughout the study period, as detailed in the study design section that follows.

### Classification of Severe, Critical, and Fatal COVID-19 According to WHO Criteria

To ensure a rigorous and standardized assessment of COVID-19 infection severity, the national protocol stipulated that every hospitalization associated with SARS-CoV-2 infection, whether in acute care or ICU beds, undergo a severity assessment based on WHO guidelines for assessing COVID-19 severity [12], criticality [12], and fatality [13], every 3 days until discharge or death [16]. This protocol was applied consistently, regardless of the length of the hospital stay, from the onset of the pandemic to the present [16].

Trained medical personnel, independent of the study investigators and the clinical teams responsible for patient care, conducted the classification of cases as severe [12], critical [12], or fatal [13] COVID-19 through individual chart reviews [16]. A detailed description of this process is provided in Supplementary Section S2. Severe cases were generally managed in acute care beds, though they were often placed in ICU beds as a precaution, whereas critical cases were always managed in ICU beds [16].

### Study Design

This nationwide observational study evaluated the diagnostic accuracy of acute-care and ICU hospitalizations associated with SARS-CoV-2 infection as indicators of infection severity, using the WHO criteria for COVID-19 severity as the reference standard [12, 13]. Two hospitalization indicators of infection severity were assessed. The first indicator was any SARS-CoV-2–associated hospitalization in either acute care or ICU beds as an indicator of severe [12], critical [12], or fatal [13] COVID-19 per WHO criteria. The second indicator was SARS-CoV-2–associated hospitalization exclusively in ICU beds as an indicator of severe [12], critical [12], or fatal [13] COVID-19 per WHO criteria.

Individual-level records of SARS-CoV-2-associated hospitalizations became available to the study investigators from 6 September 2021, which established the study's start date. All confirmed SARS-CoV-2 infections during the study period were included in the analysis. A confirmed SARS-CoV-2 infection was defined as a documented PCR- or RA-positive test, provided there had not been a previous positive test within the preceding 90 days. The 90-day interval was chosen to avoid misclassifying prolonged test positivity as a new infection, which could occur with shorter intervals [23, 24].

Each confirmed infection was reviewed for the presence of any associated hospitalization record, regardless of the cause, as well as for a record of a severity assessment according to WHO criteria. Confirmed SARS-CoV-2 infections for individuals who had a hospitalization record, whether in an acute care or ICU bed, within 5 days before or up to 14 days after the positive SARS-CoV-2 test were classified as SARS-CoV-2–associated hospitalization cases. The 5-day window before the positive test was included to account for the possibility that testing was conducted after the individual had already been hospitalized. If multiple admissions occurred within this period, such as an initial acute care admission followed by ICU transfer, the highest level of care (ie, ICU) was recorded as the admission status.

Confirmed SARS-CoV-2 infections for individuals who had a WHO severity assessment indicating severe, critical, or fatal COVID-19 within 5 days before or up to 90 days after the positive SARS-CoV-2 test were classified as severe [12], critical [12], or fatal [13] COVID-19 cases. The 90-day window was applied to capture all severe outcomes because some hospitalized patients only progressed to severe outcomes well after initial diagnosis and hospitalization.

## STATISTICAL ANALYSIS

The study sample was described using frequency distributions and measures of central tendency, with results cross-tabulated for comparison. Using the WHO criteria for COVID-19 case severity as the reference standard [12, 13], 2 concordance measures were calculated for the SARS-CoV-2–associated hospitalization indicators: overall percent agreement and Cohen's kappa statistic.

The overall percent agreement was defined as the proportion of cases in which the considered hospitalization indicator agreed with the WHO criteria, divided by the total number of SARS-CoV-2 infections. Although this metric is straightforward to calculate, it produces misleadingly high values in this study because of the predominance of mild or asymptomatic infections that are not associated with hospitalization. Nevertheless, the overall percent agreement was reported for completeness, as it is a standard measure of agreement.

In contrast, Cohen's kappa statistic provides a more robust and nuanced assessment of agreement, particularly in studies like this, where class imbalances and chance agreement can distort the reliability of the overall percent agreement [25]. Cohen's kappa measures agreement beyond what would be expected by chance, with values ranging from 0 to 1 [25]. According to convention, Cohen's kappa values greater than 0.75 indicate excellent agreement, values between 0.40 and 0.75 indicate fair to good agreement, and values below 0.40 indicate poor agreement [25]. Cohen's kappa and its 95% confidence interval (CI) were calculated using the *kap* and *kapci* commands in Stata 18.0.

Additional diagnostic performance metrics were calculated, including sensitivity, specificity, positive predictive value, and negative predictive value of the SARS-CoV-2–associated hospitalization indicators, with reference to the WHO criteria for COVID-19 severity [12, 13]. These calculations were performed using the *diagt* command in Stata 18.0.

Three sensitivity analyses were conducted to evaluate temporal variations and potential biases: stratification by pre- and post-Omicron periods (before and after 19 December 2021 [26]), stratification by testing policy changes (before and after 1 November 2022 [27]), and assessment of the impact of misclassified severe, critical, or fatal COVID-19 cases. Description of these analyses can be found in Supplementary Section S5.

All analyses were conducted using STATA/SE version 18.0 (StataCorp, College Station, TX, USA).

## ETHICS STATEMENT

The institutional review boards at Hamad Medical Corporation and Weill Cornell Medicine–Qatar approved this study with a waiver of informed consent. The study was reported in accordance with the Standards for Reporting Diagnostic Accuracy Studies guidelines (Supplementary Table 1).

## RESULTS

### Study Sample

**Figure 1.**
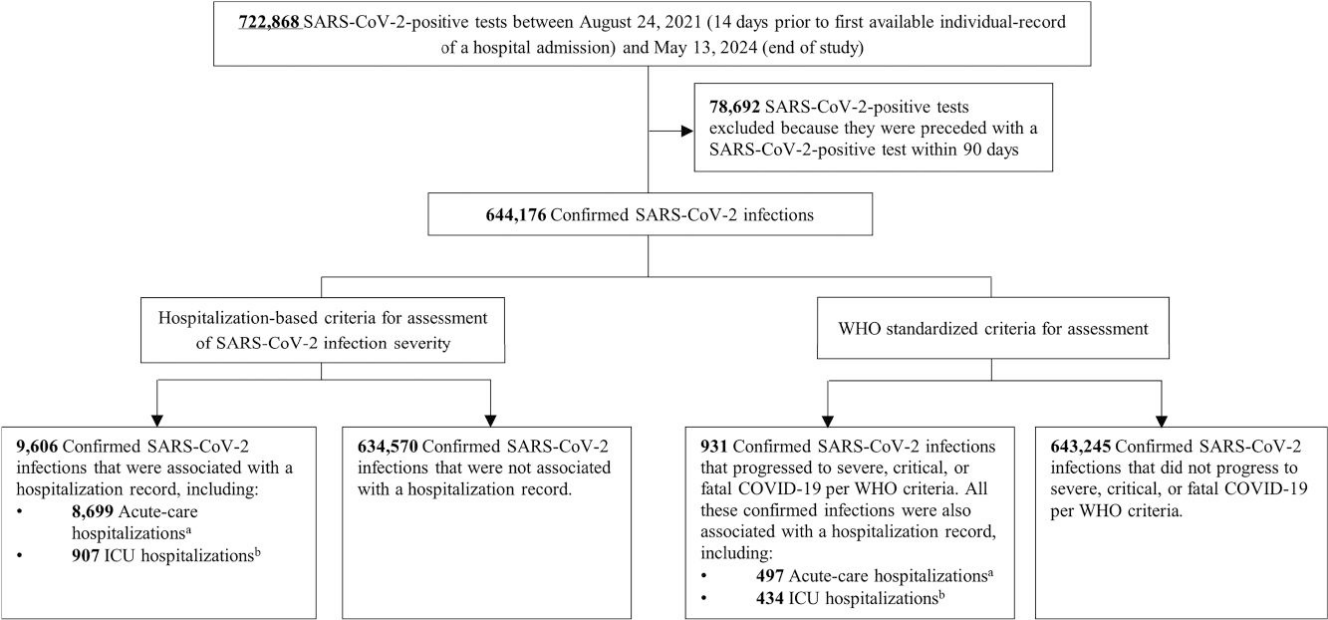
Flowchart illustrating the study sample selection process for assessing the diagnostic accuracy of acute care and ICU hospitalizations associated with SARS-CoV-2 infection as indicators of infection severity, compared to the WHO criteria for COVID-19 severity as the reference standard. ICU, intensive care unit; WHO, World Health Organization. ^a^A SARS-CoV-2–associated acute care hospital admission refers to the admission of an individual with an active SARS-CoV-2 infection to an acute care bed, regardless of the individual's clinical condition. ^b^A SARS-CoV-2–associated ICU admission refers to the admission of an individual with an active SARS-CoV-2 infection to an ICU bed, where the ICU admission clinical team determined that the condition may be related to COVID-19.

Figure 1 illustrates the study's sample selection process. The study was conducted on the entire population of Qatar, making it representative of the country's internationally diverse, yet predominantly young, demographic.

The study sample, consisting of 644 176 confirmed SARS-CoV-2 infections, had a median age of 33 years (interquartile range, 22–41) (Table 1). The largest age group was 30–39 years (30.3%), and only 11.5% of the sample was older than 50 years. Males accounted for a slight majority of the population (53.9%) compared to females (46.1%). Qatari nationals constituted 26.1% of the sample, whereas other major groups included Indian nationals (18.5%) and Filipino nationals (10.8%), reflecting the country's multicultural composition.

**Table 1.** Characteristics of the Study Sample Used to Assess the Diagnostic Accuracy of Acute Care and ICU Hospitalizations Associated With SARS-CoV-2 Infection as Indicators of Infection Severity, Using the WHO Criteria for COVID-19 Severity as the Reference Standard

Characteristics	N	%
Total	644 176	100.0%
Age group—y	…	…
0–9	68 318	10.6
10–19	71 455	11.1
20–29	123 497	19.2
30–39	195 310	30.3
40–49	111 963	17.4
50–59	48 776	7.6
60–69	17 436	2.7
≥70	7421	1.2
Median age (IQR), y	33 (22–41)	−
Sex	…	…
Male	347 135	53.9
Female	297 041	46.1
Nationality^a^	…	…
Bangladeshi	19 831	3.1
Egyptian	33 653	5.2
Filipino	69 407	10.8
Indian	118 930	18.5
Nepalese	23 658	3.7
Pakistani	22 059	3.4
Qatari	167 934	26.1
Sri Lankan	12 998	2.0
Sudanese	17 117	2.7
Other nationalities^b^	158 589	24.6
Coexisting conditions	…	…
0	479 275	74.4
1	89 033	13.8
2	37 363	5.8
3	16 662	2.6
4	9502	1.5
5	5792	0.9
≥6	6549	1.0
Reason for testing	…	…
Clinical suspicion	123 334	19.1
Survey	37 986	5.9
Contact tracing	64 505	10.0
Pretravel	95 439	14.8
Port of entry	58 864	9.1
Individual request	44 542	6.9
Healthcare routine testing	13 009	2.0
Other	970	0.2
Missing	205 527	31.9

Abbreviations: IQR, interquartile range; WHO, World Health Organization.

^a^Nationalities were chosen to represent the most populous groups in Qatar.

^b^These comprise 170 other nationalities in Qatar.

Most participants (74.4%) had no coexisting conditions, 13.8% had 1, and 11.8% had 2 or more. The primary reasons for SARS-CoV-2 testing included clinical suspicion (19.1%), pretravel requirements (14.8%), and contact tracing (10.0%), although 31.9% of the sample had missing data regarding the reason for testing.

Of the 644 176 confirmed SARS-CoV-2 infections in the study sample, 9606 were associated with a hospitalization record in either acute care or ICU beds, whereas 634 570 had no associated hospitalization record (Figure 1). Within this same study sample, 931 infections progressed to severe, critical, or fatal COVID-19 according to WHO criteria, whereas 643 245 did not.

Of the 9606 confirmed SARS-CoV-2 infections associated with hospitalization, 8699 were hospitalized in acute care beds and 907 were hospitalized in ICU beds (Figure 1). Of the 931 confirmed SARS-CoV-2 infections that progressed to severe, critical, or fatal COVID-19 per WHO criteria, 497 were hospitalized in acute care beds, whereas 434 were hospitalized in ICU beds.

### Any SARS-CoV-2–associated Hospitalization as an Indicator of Infection Severity

The cross-tabulation comparing the diagnostic performance of any SARS-CoV-2–associated hospitalization, whether in acute care or ICU beds, as an indicator of severe, critical, or fatal COVID-19 per WHO criteria is shown in Table 2A.

**Table 2.** Cross-tabulation Comparing the Diagnostic Performance of (A) any SARS-CoV-2–associated Hospitalization in Either Acute Care or ICU Beds as an Indicator of Severe, Critical, or Fatal COVID-19 per WHO Criteria and (B) SARS-CoV-2–associated Hospitalization Exclusively in ICU Beds as an Indicator of Severe, Critical, or Fatal COVID-19 per WHO Criteri

**A)**		Confirmed SARS-CoV-2 infections^a^ that progressed to severe, critical, or fatal COVID-19 per WHO criteria		
		Yes	No	Total
Confirmed SARS-CoV-2 infections^a^ that were associated with a hospitalization record in either acute care^b^ or ICU^c^ beds	Yes	931	8675	9606
	No	0	634 570	634 570
	Total	931	643 245	644 176
**B)**		Confirmed SARS-CoV-2 infections^a^ that progressed to severe, critical, or fatal COVID-19 per WHO criteria		
		Yes	No	Total
Confirmed SARS-CoV-2 infections^a^ that were associated with a hospitalization record exclusively in ICU beds^c^	Yes	434	473	907
	No	497	642 772	643 269
	Total	931	643 245	644 176

Abbreviations: ICU, intensive care unit; PCR, polymerase chain reaction; RA, rapid antigen; WHO, World Health Organization.

^a^A confirmed SARS-CoV-2 infection was defined as a documented PCR- or RA-positive test, provided there had not been a previous positive test within the preceding 90 days.

^b^A SARS-CoV-2–associated acute care hospital admission refers to the admission of an individual with an active SARS-CoV-2 infection to an acute care bed, regardless of the individual's clinical condition.

^c^A SARS-CoV-2–associated ICU admission refers to the admission of an individual with an active SARS-CoV-2 infection to an ICU bed, where the ICU admission clinical team determined that the condition may be related to COVID-19.

Table 3A presents the diagnostic performance metrics for this hospitalization indicator. The overall percent agreement between this indicator and WHO criteria was 98.7% (95% CI, 98.6–98.7). However, Cohen's kappa was only 0.17 (95% CI, 0.16–0.18), indicating poor agreement between this indicator and the WHO criteria.

**Table 3.** Diagnostic Performance Metrics for A) any SARS-CoV-2–associated Hospitalization in Either Acute Care or ICU Beds as an Indicator of Severe, Critical, or Fatal COVID-19 per WHO Criteria and B) SARS-CoV-2-associated Hospitalization Exclusively in ICU Beds as an Indicator of Severe, Critical, or Fatal COVID-19 per WHO Criteria

Reference Standard	Type of SARS-CoV-2–associated Hospital Admission	Diagnostic Concordance Assessment % (95% CI)		Diagnsotic Performance Assessment % (95% CI)			
		Overall Percent Agreement	Cohen's Kappa Statistic	Sensitivity	Specificity	Positive Predicive Value	Negative Predicive Value
WHO criteria for COVID-19 severity, criticality, and fatality	A) Hospitalization in either acute care^a^ or ICU^b^ beds	98.7 (98.6–98.7)	0.17 (0.16–0.18)	100 (99.6–100)	98.7 (98.6–98.7)	9.7 (9.1–10.3)	100 (100–100)
	B) Hospitalization exclusively in ICU beds^b^	99.8 (99.8–99.9)	0.47 (0.44–0.50)	46.6 (43.4–49.9)	99.9 (99.9–99.9)	47.9 (44.6–51.2)	99.9 (99.9–99.9)

Abbreviations: CI, confidence interval, ICU, intensive care unit; WHO, World Health Organization.

^a^A SARS-CoV-2–associated acute care hospital admission refers to the admission of an individual with an active SARS-CoV-2 infection to an acute care bed, regardless of the individual's clinical condition.

^b^A SARS-CoV-2–associated ICU admission refers to the admission of an individual with an active SARS-CoV-2 infection to an ICU bed, where the ICU admission clinical team determined that the condition may be related to COVID-19.

The sensitivity of this indicator for detecting true severity per WHO criteria was 100% (95% CI, 99.6–100), and the specificity was 98.7% (95% CI, 98.6–98.7) (Table 3A). The positive predictive value was only 9.7% (95% CI, 9.1–10.3), but the negative predictive value was 100% (95% CI, 100–100).

### SARS-CoV-2–associated Hospitalization in ICU Beds as an Indicator of Infection Severity

The cross-tabulation comparing the diagnostic performance of SARS-CoV-2–associated hospitalization exclusively in ICU beds as an indicator of severe, critical, or fatal COVID-19 per WHO criteria is shown in Table 2B.

Table 3B presents the diagnostic performance metrics for this hospitalization indicator. The overall percent agreement between this indicator and WHO criteria was 99.8% (95% CI, 99.8–99.9). Cohen's kappa was 0.47 (95% CI, 0.44–0.50), indicating fair to good agreement between this indicator and the WHO criteria.

The sensitivity of this indicator for detecting true severity per WHO criteria was 46.6% (95% CI, 43.4–49.9), and the specificity was 99.9% (95% CI, 99.9–99.9) (Table 3B). The positive predictive value was 47.9% (95% CI, 44.6–51.2), and the negative predictive value was 99.9% (95% CI, 99.9–99.9).

### Sensitivity Analyses

The results of the sensitivity analyses are presented in Supplementary Table 2, Table 4, and Supplementary Table 3, with descriptions in Supplementary Section S5. The analyses showed that the diagnostic performance of any SARS-CoV-2–associated hospitalization as an indicator of severe, critical, or fatal COVID-19 was markedly better in the pre-Omicron period and before testing policy changes than in the post-Omicron period and after policy changes (Table 4). In contrast, the diagnostic performance of ICU-only hospitalizations exhibited less variation across these periods. The analysis of misclassified cases produced results consistent with the main analysis, confirming the study findings (Supplementary Table 3).

**Table 4.** Sensitivity Analyses. Study Outcomes Stratified by Pre- and Post-Omicron Periods, as Well as Before and After Major Testing Policy Changes

Analyses	Type of SARS-CoV-2–associated Hospital Admission	Diagnostic Concordance Assessment % (95% CI)		Diagnsotic Performance Assessment % (95% CI)			
		Overall Percent Agreement	Cohen's Kappa Statistic	Sensitivity	Specificity	Positive Predicive Value	Negative Predicive Value
Analyses of pre- and post-Omicron periods^a^							
Pre-Omicron period	A) Hospitalization in either acute care^b^ or ICU^c^ beds	97.9 (97.8–98.1)	0.46 (0.43–0.50)	100 (98.6–100)	97.9 (97.8–98.1)	30.8 (27.7–34.0)	100 (100–100)
	B) Hospitalization exclusively in ICU beds^c^	99.2 (99.1–99.3)	0.53 (0.47–0.58)	48.1 (41.9–54.3)	99.7 (99.6–99.8)	59.6 (52.7–66.3)	99.5 (99.4–99.6)
Omicron period	A) Hospitalization in either acute care^b^ or ICU^c^ beds	98.7 (98.7–98.7)	0.14 (0.13–0.15)	100 (99.4–100)	98.7 (98.7–98.7)	7.6 (7.1–8.2)	100 (100–100)
	B) Hospitalization exclusively in ICU beds^c^	99.9 (99.9–99.9)	0.45 (0.42–0.48)	46.0 (42.2–49.9)	99.9 (99.9–99.9)	44.2 (40.5–48.0)	99.9 (99.9–99.9)
Analyses before and after major testing policy changes^d^							
Before major testing policy changes	A) Hospitalization in either acute care^b^ or ICU^c^ beds	99.0 (98.9–99.0)	0.21 (0.20–0.22)	100 (99.5–100)	99.0 (98.9–99.0)	11.9 (11.1–12.7)	100 (100–100)
	B) Hospitalization exclusively in ICU beds^c^	99.9 (99.9–99.9)	0.50 (0.47–0.53)	48.9 (45.4–52.5)	99.9 (99.9–99.9)	51.0 (47.4–54.6)	99.9 (99.9–99.9)
After major testing policy changes	A) Hospitalization in either acute care^b^ or ICU^c^ beds	96.7 (96.6–96.8)	0.09 (0.08–0.10)	100 (97.4–100)	96.7 (96.6–96.8)	4.8 (4.0–5.6)	100 (100–100)
	B) Hospitalization exclusively in ICU beds^c^	99.8 (99.7–99.8)	0.33 (0.26–0.40)	33.8 (26.1–42.2)	99.9 (99.9–99.9)	32.0 (24.6–40.1)	99.9 (99.9–99.9)

Diagnostic performance metrics for A) any SARS-CoV-2–associated hospitalization in either acute care or ICU beds as an indicator of severe, critical, or fatal COVID-19 per WHO criteria and B) SARS-CoV-2–associated hospitalization exclusively in ICU beds as an indicator of severe, critical, or fatal COVID-19 per WHO criteria.

Abbreviations: CI, confidence interval; ICU, intensive care unit; WHO, World Health Organization.

^a^The Omicron period began on 19 December 2021, marking the onset of the first Omicron wave.

^b^A SARS-CoV-2–associated acute care hospital admission refers to the admission of an individual with an active SARS-CoV-2 infection to an acute care bed, regardless of the individual's clinical condition.

^c^A SARS-CoV-2–associated ICU admission refers to the admission of an individual with an active SARS-CoV-2 infection to an ICU bed, where the ICU admission clinical team determined that the condition may be related to COVID-19.

^d^The major changes to testing policies were introduced on 1 November 2022.

## DISCUSSION

The study identified a mismatch between any hospitalizations associated with a positive SARS-CoV-2 test and the true severity of COVID-19, as evidenced by a very low positive predictive value (<10%) and a Cohen's kappa of only 0.17. This indicates that the majority of these hospitalizations did not involve severe COVID-19. In many cases, the positive SARS-CoV-2 test was incidental, detected during hospital admissions for unrelated conditions (hospitalization with COVID-19 rather than because of COVID-19). Other contributing factors may have included precautionary hospitalization due to underlying health concerns or advanced age, worsening preexisting conditions, isolation protocols, difficulty in managing symptoms at home, or cases where COVID-19 was present but not severe enough to meet the WHO criteria for severity.

The mismatch was particularly evident during the Omicron era, as demonstrated by the sensitivity analyses. This discrepancy can be attributed to the lower severity of Omicron subvariants compared to earlier variants [28–32], accumulation of vaccine-induced population immunity [18, 20, 33], and buildup of infection-induced immunity [16, 33–35]. The forward displacement of deaths among individuals with relatively short life expectancy may have also contributed [21]. Together, these factors reduced the progression of infections to severe disease over time, thereby decreasing the reliability of general hospitalizations as an indicator of COVID-19 severity.

These findings underscore that generic hospital admission data should not be used as a proxy for COVID-19 severity because its use will likely introduce bias in estimating epidemiologic indicators, such as the true severity of new variants. It will likely also lead to an underestimation of the protective effects of vaccination and natural immunity in preventing severe infections.

Misclassifying severity based solely on hospitalization records can affect the outcomes of vaccine effectiveness studies, as effectiveness would be assessed against a less severe outcome, where effectiveness is lower [10, 18, 36, 37]. Similarly, this concern extends to studies evaluating protection conferred by infection against reinfection because protection is lower for less severe outcomes [38, 39]. However, for health system assessments of bed utilization and staffing requirements, this misclassification may hold less significance.

This conclusion aligns with patterns observed in vaccine effectiveness studies, in which the use of specific indicators for COVID-19 severity—such as WHO criteria, oxygen use, mechanical ventilation, or ICU admission due to respiratory symptoms—yields higher estimates of effectiveness compared to studies relying on generic hospital admission data [14, 15, 18]. Notably, this issue has gained increasing recognition, with recent effectiveness studies involving hospitalized patients incorporating clinical criteria related to respiratory disease and/or severity (eg, a minimum hospital stay of 2 days) rather than relying exclusively on hospitalization status, as was common earlier [10, 14, 15, 40].

Although generic hospital admissions were poor indicators of COVID-19 severity, ICU-only SARS-CoV-2–associated hospitalizations provided a more reasonable, though imperfect, measure, as shown in the main and sensitivity analyses. This is supported by a moderate Cohen's kappa of 0.47, indicating fair to good agreement with true severity. The positive predictive value of 47.9% showed that nearly half of ICU hospitalizations involved true severe cases, though a sensitivity of 46.6% revealed that more than half of true severe cases were missed.

In light of these findings, COVID-19 severity assessments should be based on rigorous criteria, such as WHO criteria. If this is not feasible because of resource constraints, assessments should, at a minimum, rely on ICU admissions for respiratory symptoms or hospitalization records indicating oxygen use or mechanical ventilation [12–15].

This study has limitations. The findings are specific to Qatar's population, which may limit their generalizability to other countries. Qatar, with its high human development index and well-resourced healthcare system [16], maintained a low threshold for hospitalization during the pandemic [2, 16, 17]. This may have contributed to the poor performance of hospitalization as an indicator of COVID-19 severity.

Milder cases requiring hospitalization but not captured by WHO criteria may also have contributed to the poor performance of hospitalization as a severity indicator. The study began after vaccines and treatments were introduced [11, 18, 20], missing the earlier pandemic phase when hospitalization was a more reliable proxy for severity. Although the study classified severe, critical, and fatal COVID-19 cases based on WHO criteria [12, 13], the spectrum of severity evolved over time because of viral evolution and increasing population immunity [16, 41, 42].

In the early pandemic, severe COVID-19 primarily resulted from pulmonary disease caused by the virus's direct impact on the lungs [29, 30, 43]. With the emergence of Omicron, reduced lung tropism led to fewer pneumonia cases [29–32, 43], and many hospitalizations were due to exacerbations of underlying conditions rather than direct viral effects [29]. The WHO criteria, designed for the original virus, may not fully capture the evolving severity spectrum.

SARS-CoV-2–associated hospitalizations can be defined in various ways, each incorporating different considerations, and the performance of these definitions as indicators of true COVID-19 severity may vary. Countries have adopted differing definitions, influenced by the structure and capabilities of their digital health platforms and how these systems are utilized to identify and classify hospitalizations [10, 14, 15, 40]. Moreover, these definitions have evolved over time, becoming more specific and refined as the pandemic progressed [14, 15, 40].

SARS-CoV-2–associated hospitalizations in Qatar were identified based on a national protocol, with the definition of active infection evolving over time [16]. This study used a consistent, modified definition throughout. Although this mismatch may have led to missed hospitalizations, it did not affect the classification of severe, critical, or fatal COVID-19 cases per WHO criteria, which followed a separate protocol. Consequently, the diagnostic metrics for the hospitalization indicators may appear more favorable than they truly are, further supporting the study's conclusion about the limitations of SARS-CoV-2–associated hospitalizations as indicators of COVID-19 severity.

The study is based on documented SARS-CoV-2 infections; however, some may have gone undocumented or been diagnosed using home rapid antigen tests not recorded in national databases [35, 39]. These undocumented infections, typically mild or asymptomatic, are unlikely to affect the study results, as they have minimal impact on the diagnostic performance metrics comparing hospitalization indicators to WHO severity criteria. Additionally, given case management protocols, it is unlikely that hospitalized patients who initially tested positive at home were admitted without confirmatory testing at the hospital or additional testing as part of standard care.

The study was a retrospective analysis of real-world data derived from documented cases within Qatar's healthcare system. Although it cannot be guaranteed that all hospitalized or severe cases were captured in such real-world data, the methods for case ascertainment were rigorous, detailed, and comprehensive. Additionally, Qatar's healthcare system was not resource-constrained at any point during the pandemic, partly because of the relatively lower severity of the pandemic in its predominantly young population [16, 44]. These factors suggest that even if some cases were missed or misclassified, their numbers would be negligible and unlikely to materially impact the results or alter the conclusions of this study.

The study has strengths. The study was conducted in a country that employed a rigorous approach to the identification of severe, critical, and fatal COVID-19 cases throughout the pandemic, adhering strictly to the WHO criteria [12, 13]. This case ascertainment was carried out by trained medical personnel following a national protocol applied consistently to all hospitalized COVID-19 cases in Qatar. The study was conducted on a national scale, encompassing a diverse population with a wide range of national backgrounds, and leveraged extensive, validated databases established through numerous SARS-CoV-2 infection studies.

In conclusion, this study highlights the limitations of using generic hospital admission data as a proxy for COVID-19 severity, particularly at this stage of the pandemic. Most hospitalizations associated with a positive SARS-CoV-2 test are not due to severe COVID-19, rendering such data unreliable for assessing disease severity. However, ICU hospitalizations, although not a perfect indicator, are more reliable. The findings also underscore the importance of using specific, robust criteria—such as those of the WHO criteria—for evaluating COVID-19 severity. When resource constraints make this challenging, ICU admissions for respiratory symptoms or records of oxygen use or mechanical ventilation can serve as alternative indicators to minimize bias in epidemiological assessments and vaccine effectiveness studies.

## Supplementary Material

ofaf098_Supplementary_Data

## Supplementary Data

Supplementary materials are available at *Open Forum Infectious Diseases* online. Consisting of data provided by the authors to benefit the reader, the posted materials are not copyedited and are the sole responsibility of the authors, so questions or comments should be addressed to the corresponding author.

## Notes

***Acknowledgments.*** The authors acknowledge the many dedicated individuals at Hamad Medical Corporation, the Ministry of Public Health, the Primary Health Care Corporation, Qatar Biobank, Sidra Medicine, and Weill Cornell Medicine-Qatar for their diligent efforts and contributions to make this study possible. The authors are also grateful for institutional salary support from the Biomedical Research Program and the Biostatistics, Epidemiology, and Biomathematics Research Core, both at Weill Cornell Medicine-Qatar, as well as for institutional salary support provided by the Ministry of Public Health, Hamad Medical Corporation, and Sidra Medicine. The authors are also grateful for the Qatar Genome Programme and Qatar University Biomedical Research Center for institutional support for the reagents needed for the viral genome sequencing. ChatGPT was exclusively utilized to verify grammar and refine the English phrasing in our text. No other functionalities or applications of ChatGPT were employed beyond this specific scope. Following the use of this tool, the authors thoroughly reviewed and edited the content as necessary and take full responsibility for the accuracy and quality of the publication.

***Author Contributions.*** L. S. codesigned the study, performed the statistical analyses, and cowrote the first draft of the article. L. J. A. conceived and codesigned the study, led the statistical analyses, and cowrote the first draft of the article. H. C. codesigned the study and performed statistical analyses. L. S., H. C., and L. J. A. accessed and verified all the data. P. V. C. designed mass polymerase chain reaction testing to allow routine capture of variants and conducted viral genome sequencing. P. T. and M. R. H. designed and conducted multiplex, real-time quantitative polymerase chain reaction variant screening, and viral genome sequencing. H. M. Y. and A. A. A. T. conducted viral genome sequencing. All authors contributed to data collection and acquisition, database development, discussion and interpretation of the results, and to the writing of the article. All authors have read and approved the final manuscript.

***Data availability.*** The dataset of this study is a property of the Qatar Ministry of Public Health that was provided to the researchers through a restricted-access agreement that prevents sharing the dataset with a third party or publicly. The data are available under restricted access for preservation of confidentiality of patient data. Access can be obtained through a direct application for data access to Her Excellency the Minister of Public Health (https://www.moph.gov.qa/english/OurServices/eservices/Pages/Governmental-Health-Communication-Center.aspx). The raw data are protected and are not available because of data privacy laws. Aggregate data are available within the paper and its supplementary information.

***Financial support.*** This work was supported by the Biomedical Research Program and the Biostatistics, Epidemiology, and the Biomathematics Research Core, both at Weill Cornell Medicine-Qatar, as well as the Ministry of Public Health, Hamad Medical Corporation, Sidra Medicine, Qatar Genome Programme, and Qatar University Biomedical Research Center. The funders of the study had no role in the study design; in the collection, analysis, and interpretation of data; in the writing of the report; and in the decision to submit the article for publication. Statements made herein are solely the responsibility of the authors.

## References

[1] Msemburi W, Karlinsky A, Knutson V, Aleshin-Guendel S, Chatterji S, Wakefield J. The WHO estimates of excess mortality associated with the COVID-19 pandemic. Nature 2023; 613:130–7. 36517599 10.1038/s41586-022-05522-2 PMC9812776

[2] Seedat S, Chemaitelly H, Ayoub HH, et al SARS-CoV-2 infection hospitalization, severity, criticality, and fatality rates in Qatar. Sci Rep 2021; 11:18182. 34521903 10.1038/s41598-021-97606-8 PMC8440606

[3] Ayoub HH, Mumtaz GR, Seedat S, Makhoul M, Chemaitelly H, Abu-Raddad LJ. Estimates of global SARS-CoV-2 infection exposure, infection morbidity, and infection mortality rates in 2020. Glob Epidemiol 2021; 3:100068. 34841244 10.1016/j.gloepi.2021.100068 PMC8609676

[4] Abu-Raddad LJ, Chemaitelly H, Ayoub HH, et al Severity, criticality, and fatality of the severe acute respiratory syndrome coronavirus 2 (SARS-CoV-2) Beta variant. Clin Infect Dis 2022; 75:e1188–91. 34657152 10.1093/cid/ciab909 PMC9402694

[5] Flaxman S, Mishra S, Gandy A, et al Estimating the effects of non-pharmaceutical interventions on COVID-19 in Europe. Nature. 2020; 584:257–61. 32512579 10.1038/s41586-020-2405-7

[6] Makhoul M, Ayoub HH, Chemaitelly H, et al Epidemiological impact of SARS-CoV-2 vaccination: mathematical modeling analyses. Vaccines (Basel) 2020; 8:668. 33182403 10.3390/vaccines8040668 PMC7712303

[7] Polack FP, Thomas SJ, Kitchin N, et al Safety and efficacy of the BNT162b2 mRNA COVID-19 vaccine. N Engl J Med 2020; 383:2603–15. 33301246 10.1056/NEJMoa2034577 PMC7745181

[8] Makhoul M, Abu-Hijleh F, Ayoub HH, Seedat S, Chemaitelly H, Abu-Raddad LJ. Modeling the population-level impact of treatment on COVID-19 disease and SARS-CoV-2 transmission. Epidemics 2022; 39:100567. 35468531 10.1016/j.epidem.2022.100567 PMC9013049

[9] Recovery Collaborative Group, Horby P, Lim WS, et al Dexamethasone in hospitalized patients with COVID-19. N Engl J Med. 2021; 384:693–704. 32678530 10.1056/NEJMoa2021436 PMC7383595

[10] Feikin DR, Higdon MM, Abu-Raddad LJ, et al Duration of effectiveness of vaccines against SARS-CoV-2 infection and COVID-19 disease: results of a systematic review and meta-regression. Lancet 2022; 399:924–44. 35202601 10.1016/S0140-6736(22)00152-0 PMC8863502

[11] Zaqout A, Almaslamani MA, Chemaitelly H, et al Effectiveness of the neutralizing antibody sotrovimab among high-risk patients with mild-to-moderate SARS-CoV-2 in Qatar. Int J Infect Dis 2022; 124:96–103. 36218031 10.1016/j.ijid.2022.09.023 PMC9484101

[12] World Health Organization (WHO) . Living guidance for clinical management of COVID-19. Available at: https://www.who.int/publications/i/item/WHO-2019-nCoV-clinical-2021-2. Accessed on: 27 February 2023.

[13] World Health Organization (WHO) . International Guidelines for Certification and Classification (Coding) of COVID-19 as Cause of Death. Available at: https://www.who.int/publications/m/item/international-guidelines-for-certification-and-classification-(coding)-of-covid-19-as-cause-of-death. Accessed on: 27 February 2023.

[14] Feikin DR, Abu-Raddad LJ, Andrews N, et al Assessing vaccine effectiveness against severe COVID-19 disease caused by omicron variant. Report from a meeting of the World Health Organization. Vaccine 2022; 40:3516–27. 35595662 10.1016/j.vaccine.2022.04.069 PMC9058052

[15] Stowe J, Andrews N, Kirsebom F, Ramsay M, Bernal JL. Effectiveness of COVID-19 vaccines against Omicron and Delta hospitalisation, a test negative case-control study. Nat Commun 2022; 13:5736. 36180428 10.1038/s41467-022-33378-7 PMC9523190

[16] Chemaitelly H, Ayoub HH, Faust JS, et al Turning point in COVID-19 severity and fatality during the pandemic: a national cohort study in Qatar. BMJ Public Health 2023; 1:e000479. 40017867 10.1136/bmjph-2023-000479 PMC11812731

[17] Abu-Raddad LJ, Chemaitelly H, Ayoub HH, et al Characterizing the Qatar advanced-phase SARS-CoV-2 epidemic. Sci Rep 2021; 11:6233. 33737535 10.1038/s41598-021-85428-7 PMC7973743

[18] Chemaitelly H, Tang P, Hasan MR, et al Waning of BNT162b2 vaccine protection against SARS-CoV-2 infection in Qatar. N Engl J Med 2021; 385:e83. 34614327 10.1056/NEJMoa2114114 PMC8522799

[19] Altarawneh HN, Chemaitelly H, Ayoub HH, et al Effects of previous infection and vaccination on symptomatic Omicron infections. N Engl J Med 2022; 387:21–34. 35704396 10.1056/NEJMoa2203965 PMC9258753

[20] Abu-Raddad LJ, Chemaitelly H, Ayoub HH, et al Effect of mRNA vaccine boosters against SARS-CoV-2 Omicron infection in Qatar. N Engl J Med 2022; 386:1804–16. 35263534 10.1056/NEJMoa2200797 PMC8929389

[21] Chemaitelly H, Faust JS, Krumholz HM, et al Short- and longer-term all-cause mortality among SARS-CoV-2-infected individuals and the pull-forward phenomenon in Qatar: a national cohort study. Int J Infect Dis 2023; 136:81–90. 37717648 10.1016/j.ijid.2023.09.005

[22] Chemaitelly H, Ayoub HH, Tang P, et al Long-term COVID-19 booster effectiveness by infection history and clinical vulnerability and immune imprinting: a retrospective population-based cohort study. Lancet Infect Dis 2023; 23:816–27. 36913963 10.1016/S1473-3099(23)00058-0 PMC10079373

[23] Pilz S, Theiler-Schwetz V, Trummer C, Krause R, Ioannidis JPA. SARS-CoV-2 reinfections: overview of efficacy and duration of natural and hybrid immunity. Environ Res 2022; 209:112911. 35149106 10.1016/j.envres.2022.112911 PMC8824301

[24] Chemaitelly H, Ayoub HH, Tang P, et al Addressing bias in the definition of SARS-CoV-2 reinfection: implications for underestimation. Front Med (Lausanne) 2024; 11:1363045. 38529118 10.3389/fmed.2024.1363045 PMC10961414

[25] Fleiss JL, Levin B, Paik MC. The measurement of interrater agreement. Statistical methods for rates and proportions. New York: John Wiley & Sons; 2013.

[26] Altarawneh HN, Chemaitelly H, Hasan MR, et al Protection against the Omicron variant from previous SARS-CoV-2 infection. N Engl J Med 2022; 386:1288–90. 35139269 10.1056/NEJMc2200133 PMC8849180

[27] Chemaitelly H, Ayoub HH, AlMukdad S, et al Bivalent mRNA-1273.214 vaccine effectiveness against SARS-CoV-2 Omicron XBB* infections. J Travel Med 2023; 30:taad106. 37555656 10.1093/jtm/taad106 PMC10481416

[28] Butt AA, Dargham SR, Coyle P, et al COVID-19 Disease severity in persons infected with Omicron BA.1 and BA.2 sublineages and association with vaccination status. JAMA Intern Med 2022; 182:1097–9. 35994264 10.1001/jamainternmed.2022.3351 PMC9396464

[29] Hirotsu Y, Kakizaki Y, Saito A, et al Lung tropism in hospitalized patients following infection with SARS-CoV-2 variants from D614G to Omicron BA.2. Commun Med (Lond) 2023; 3:32. 36841870 10.1038/s43856-023-00261-5 PMC9959956

[30] Nyberg T, Ferguson NM, Nash SG, et al Comparative analysis of the risks of hospitalisation and death associated with SARS-CoV-2 Omicron (B.1.1.529) and Delta (B.1.617.2) variants in England: a cohort study. Lancet 2022; 399:1303–12. 35305296 10.1016/S0140-6736(22)00462-7 PMC8926413

[31] Suzuki R, Yamasoba D, Kimura I, et al Attenuated fusogenicity and pathogenicity of SARS-CoV-2 Omicron variant. Nature 2022; 603:700–5. 35104835 10.1038/s41586-022-04462-1 PMC8942852

[32] Meng B, Abdullahi A, Ferreira I, et al Altered TMPRSS2 usage by SARS-CoV-2 Omicron impacts infectivity and fusogenicity. Nature 2022; 603:706–14. 35104837 10.1038/s41586-022-04474-x PMC8942856

[33] Qassim SH, Chemaitelly H, Ayoub HH, et al Population immunity of natural infection, primary-series vaccination, and booster vaccination in Qatar during the COVID-19 pandemic: an observational study. EClinicalMedicine 2023; 62:102102. 37533414 10.1016/j.eclinm.2023.102102 PMC10393554

[34] Abu-Raddad LJ, Chemaitelly H, Bertollini R. National study group for COVID epidemiology. Severity of SARS-CoV-2 reinfections as compared with primary infections. N Engl J Med 2021; 385:2487–9. 34818474 10.1056/NEJMc2108120 PMC8631440

[35] Chemaitelly H, Nagelkerke N, Ayoub HH, et al Duration of immune protection of SARS-CoV-2 natural infection against reinfection. J Travel Med 2022; 29:taac109. 36179099 10.1093/jtm/taac109 PMC9619565

[36] Abu-Raddad LJ, Chemaitelly H, Bertollini R. National study group for COVID vaccination. Waning mRNA-1273 vaccine effectiveness against SARS-CoV-2 infection in Qatar. N Engl J Med 2022; 386:1091–3. 35081294 10.1056/NEJMc2119432 PMC8809505

[37] Sukik L, Chemaitelly H, Ayoub HH, et al Effectiveness of two and three doses of COVID-19 mRNA vaccines against infection, symptoms, and severity in the pre-Omicron era: a time-dependent gradient. Vaccine 2024; 42:3307–20. 38616439 10.1016/j.vaccine.2024.04.026

[38] Chemaitelly H, Coyle P, Ben Kacem MA, et al Protection of natural infection against reinfection with SARS-CoV-2 JN.1 variant. J Travel Med 2024; 31:taae053. 38591115 10.1093/jtm/taae053 PMC11149714

[39] Chemaitelly H, Ayoub HH, Coyle P, et al Differential protection against SARS-CoV-2 reinfection pre- and post-Omicron. Nature 2024; doi:10.1038/s41586-024-08511-9 PMC11946897 39910292

[40] VIEW-hub by IVAC . Vaccine effectiveness studies. COVID-19 data. Available at: https://view-hub.org/covid-19/effectiveness-studies. Accessed on 13 January 2025.

[41] Markov PV, Ghafari M, Beer M, et al The evolution of SARS-CoV-2. Nat Rev Microbiol 2023; 21:361–79. 37020110 10.1038/s41579-023-00878-2

[42] Subissi L, von Gottberg A, Thukral L, et al An early warning system for emerging SARS-CoV-2 variants. Nat Med 2022; 28:1110–5. 35637337 10.1038/s41591-022-01836-w PMC11346314

[43] Hammer MM, Sodickson AD, Marshall AD, Faust JS. Prevalence of pneumonia among patients who died with COVID-19 infection in ancestral versus Omicron variant eras. Acad Radiol 2024; 31:1–6. 37271637 10.1016/j.acra.2023.05.008 PMC10172968

[44] AlNuaimi AA, Chemaitelly H, Semaan S, et al All-cause and COVID-19 mortality in Qatar during the COVID-19 pandemic. BMJ Glob Health 2023; 8:e012291. 10.1136/bmjgh-2023-012291 PMC10163334 37142299

