# Supplementary material for: Evaluating Hospital Admission Data as Indicators of COVID-19 Severity: A National Assessment in Qatar
Source: Open Forum Infect Dis. 2025 Feb 17;12(3):ofaf098. doi: 10.1093/ofid/ofaf098 (PMC11909638; doi:10.1093/ofid/ofaf098)
Supplement: ofaf098_Supplementary_Data [file ofaf098_supplementary_data.docx]

**Supplementary Material**

**Table of Contents**

[**Section S1. Study population and data sources.** 2](#_Toc189314219)

[**Section S2. COVID-19 severity, criticality, and fatality classification.** 5](#_Toc189314220)

[**Severe COVID-19** 6](#_Toc189314221)

[**Critical COVID-19** 7](#_Toc189314222)

[**Fatal COVID-19** 7](#_Toc189314223)

[**Section S3. Laboratory methods.** 8](#_Toc189314224)

[**Real-time reverse-transcription polymerase chain reaction testing** 8](#_Toc189314225)

[**Rapid antigen testing** 8](#_Toc189314226)

[**Section S4. Classification of coexisting conditions.** 9](#_Toc189314227)

[**Section S5. Sensitivity analyses.** 11](#_Toc189314228)

[**Table S1. Standards for Reporting Diagnostic Accuracy Studies (STARD) checklist for diagnostic accuracy studies.** 13](#_Toc189314229)

[**Table S2. Sensitivity analyses. Study outcomes stratified by pre- and post-omicron periods, as well as before and after major testing policy changes. Cross-tabulation comparing the diagnostic performance of A) any SARS-CoV-2-associated hospitalization in either acute-care or ICU beds as an indicator of severe, critical, or fatal COVID-19 per WHO criteria, and B) SARS-CoV-2-associated hospitalization exclusively in ICU beds as an indicator of severe, critical, or fatal COVID-19 per WHO criteria.** 15](#_Toc189314230)

[**Table S3. Sensitivity analysis. The impact of misclassified severe, critical, or fatal COVID-19 cases. A) Cross-tabulation comparing the diagnostic performance of any SARS-CoV-2-associated hospitalization in either acute-care or ICU beds as an indicator of severe, critical, or fatal COVID-19 per WHO criteria. B) Diagnostic performance metrics for any SARS-CoV-2-associated hospitalization in either acute-care or ICU beds as an indicator of severe, critical, or fatal COVID-19 per WHO criteria.** 17](#_Toc189314231)

[**References** 18](#_Toc189314232)

# **Section** **S1.** **Study population and data sources.**

Qatar's national and universal public healthcare system uses the Cerner Millenium electronic medical record (EMR) system to track all the public healthcare encounters of each individual in the country, including all citizens and residents registered in the national and universal public healthcare system. Registration in the public healthcare system is mandatory for citizens and residents.

The databases analyzed in this study are data-extract downloads from the national EMR database that have been implemented on a regular weekly schedule since the onset of pandemic by the Business Intelligence Unit at Hamad Medical Corporation (HMC). HMC is the national public healthcare provider in Qatar. At every download, all severe acute respiratory syndrome coronavirus 2 (SARS-CoV-2) tests, coronavirus disease 2019 (COVID-19) vaccinations, hospitalizations related to COVID-19, and all death records regardless of cause are provided to the authors through .csv files. These databases have been analyzed throughout the pandemic not only for study-related purposes, but also to provide policymakers with summary data and analytics to inform the national response.

Every health encounter in the national EMR is linked to an individual through the HMC Number, which serves as a unique identifier that links all records for this individual at the national level. Databases were merged and analyzed using the HMC Number to link all records pertaining to testing, vaccinations, hospitalizations, and deaths. All deaths in Qatar are recorded by the public healthcare system. COVID-19-related healthcare was provided exclusively in the public healthcare system. COVID-19 vaccination was also provided only through the public healthcare system. These health records were tracked throughout the COVID-19 pandemic using the national EMR system. This system has been implemented in 2013, before the onset of the pandemic. This pre-established system ensured that we had access to comprehensive health records related to this study for both citizens and residents throughout the entire pandemic, allowing us to follow each person over time.

Demographic details for every HMC Number (individual) such as sex, age, and nationality are collected upon issuing of the universal health card, based on the Qatar Identity Card, which is a mandatory requirement by the Ministry of Interior to every citizen and resident in the country. Data extraction from the Qatar Identity Card to the digital health platform is performed electronically through scanning techniques.

SARS-CoV-2 testing in any facility in Qatar is tracked nationally in one database, the national testing database. This database covers all testing throughout the country, whether in public or private facilities. Every polymerase chain reaction (PCR) test and a proportion of the facility-based rapid antigen tests conducted in Qatar, regardless of location or setting, are classified on the basis of symptoms and the reason for testing, such as the presence of clinical symptoms, contact tracing, participation in surveys or random testing campaigns, individual requests for testing, routine healthcare testing, pre-travel requirements, at the point of entry into the country, or any other relevant reasons for testing.

Before November 1, 2022, SARS-CoV-2 testing in Qatar was performed extensively with about 5% of the population tested every week [1]. Based on the distribution of the reason for testing up to November 1, 2022, most of the tests in Qatar were conducted for routine reasons, such as travel-related purposes, and about 75% of infections were diagnosed not because of presence of symptoms [1, 2]. Starting from November 1, 2022, testing for SARS-CoV-2 was substantially reduced with <1% of the population tested every week [2]. This study factored all SARS-CoV-2-related testing included in the national testing database over the duration of the study.

December 19, 2021 marked the onset of the omicron wave in Qatar [1]. The first omicron wave that reached its peak in January of 2022 was massive and strained the testing capacity in the country [1, 3-5]. To alleviate the burden on PCR testing, rapid antigen testing was rapidly introduced. The swift change in testing policy precluded incorporating reason for testing for a number of rapid antigen tests. While the reason for testing is documented for all PCR tests, it is not uniformly available for all rapid antigen tests. However, all medically supervised rapid antigen tests were captured by the national integrated digital health platform since January 5, 2022.

Rapid antigen test kits are accessible for purchase at pharmacies in Qatar, but results of home-based testing are neither reported nor documented in the national databases. Since SARS-CoV-2-test outcomes were linked to specific public health measures, restrictions, and privileges, testing policy and guidelines stress facility-based testing as the core testing mechanism in the population. While facility-based testing is provided free of charge or at low subsidized costs, depending on the reason for testing, home-based rapid antigen testing is de-emphasized and not supported as part of national policy.

Qatar launched its COVID-19 vaccination program in December 2020, employing mRNA vaccines and prioritizing individuals based on coexisting conditions and age criteria [2, 6]. COVID-19 vaccination was provided free of charge, regardless of citizenship or residency status, and was nationally tracked [2, 6].

Qatar has unusually young, diverse demographics, in that only 9% of its residents are ≥50 years of age, and 89% are expatriates from over 150 countries [2, 7]. Further descriptions of the study population and these national databases were reported previously [1, 2, 5, 8-11].

# **Section S2. COVID-19 severity, criticality, and fatality classification.**

All COVID-19-related tertiary healthcare in Qatar was mandated to be delivered exclusively through Qatar's public healthcare system and provided free of charge to all residents. Qatar ranks among the countries with the highest per capita incomes globally, boasts a high human development index, and maintains a well-resourced healthcare system with ample bed capacity [12]. As a result, Qatar has a relatively low threshold for hospitalization, not only for COVID-19 but also for other health conditions, with healthcare services offered either free of charge or at subsidized, nominal costs through its national, universal public healthcare system.

To ensure a rigorous and standardized assessment of COVID-19 infection severity, the national protocol stipulated that every hospitalization associated with SARS-CoV-2 infection, whether in acute-care or ICU beds, undergo a severity assessment based on World Health Organization (WHO) guidelines for assessing COVID-19 severity [13], criticality [13], and fatality [14], every three days until discharge or death [12]. This protocol was applied consistently, regardless of the length of the hospital stay, from the onset of the pandemic to the present [12].

A dedicated medical team within Qatar's public healthcare system was trained at the onset of the pandemic to conduct the standardized severity classifications of COVID-19 cases based on WHO criteria [12-14]. This team was granted access to Qatar's Cerner Millennium electronic medical record system, which tracks all public healthcare encounters for every individual in the country, including both citizens and residents.

The trained personnel, independent of the study investigators and the clinical teams responsible for COVID-19 patient care, systematically classified cases as severe [13], critical [13], or fatal [14] COVID-19 according to WHO criteria. These classifications were determined based on detailed reviews of individual patient charts, evaluations of International Classification of Diseases codes and/or procedure codes, as well as other relevant encounters recorded in the electronic medical records. All classifications were documented in a national COVID-19 database, which was shared with the study investigators at regularly scheduled intervals for research purposes [12].

Severe COVID-19 cases, as defined by WHO criteria [13], were generally managed in acute-care beds, although they were often placed in ICU beds as a precaution. Critical COVID-19 cases, per WHO criteria [13], were exclusively managed in ICU beds [12].

There were 24 cases classified as COVID-19 deaths per WHO criteria [14] that were not formally hospitalized with the infection immediately prior to death. These cases were identified through admission to the national morgue, housed within the same tertiary public healthcare system responsible for all COVID-19 hospital care in Qatar. Because these individuals were admitted to the morgue within this healthcare system, had an associated positive SARS-CoV-2 test, and exhibited disease severity culminating in death, they were included in the analysis and considered to meet the case definition for an ICU hospitalization associated with a positive test.

Following our earlier studies [12, 15, 16], individuals who progressed to severe, critical, or fatal COVID-19 between the time of the documented infection and the end of the study were classified based on their worst outcome, starting with death [14], followed by critical disease [13], and then severe disease [13].

## **Severe COVID-19**

Severe COVID-19 disease was defined per WHO classification as a SARS-CoV-2 infected person with "oxygen saturation of <90% on room air, and/or respiratory rate of >30 breaths/minute in adults and children >5 years old (or ≥60 breaths/minute in children <2 months old or ≥50 breaths/minute in children 2-11 months old or ≥40 breaths/minute in children 1–5 years old), and/or signs of severe respiratory distress (accessory muscle use and inability to complete full sentences, and, in children, very severe chest wall indrawing, grunting, central cyanosis, or presence of any other general danger signs)" [13]. Detailed WHO criteria for classifying SARS-CoV-2 infection severity can be found in the WHO technical report [13].

## **Critical COVID-19**

Critical COVID-19 disease was defined per WHO classification as a SARS-CoV-2 infected person with "acute respiratory distress syndrome, sepsis, septic shock, or other conditions that would normally require the provision of life sustaining therapies such as mechanical ventilation (invasive or non-invasive) or vasopressor therapy" [13]. Detailed WHO criteria for classifying SARS-CoV-2 infection criticality can be found in the WHO technical report [13].

## **Fatal COVID-19**

COVID-19 death was defined per WHO classification as "a death resulting from a clinically compatible illness, in a probable or confirmed COVID-19 case, unless there is a clear alternative cause of death that cannot be related to COVID-19 disease (e.g. trauma). There should be no period of complete recovery from COVID-19 between illness and death. A death due to COVID-19 may not be attributed to another disease (e.g. cancer) and should be counted independently of preexisting conditions that are suspected of triggering a severe course of COVID-19". Detailed WHO criteria for classifying COVID-19 death can be found in the WHO technical report [14].

# **Section S3. Laboratory methods.**

## **Real-time reverse-transcription polymerase chain reaction testing**

Nasopharyngeal and/or oropharyngeal swabs were collected for PCR testing and placed in Universal Transport Medium (UTM). Aliquots of UTM were: 1) extracted on KingFisher Flex (Thermo Fisher Scientific, USA), MGISP-960 (MGI, China), or ExiPrep 96 Lite (Bioneer, South Korea) followed by testing with real-time reverse-transcription PCR (RT-qPCR) using TaqPath COVID-19 Combo Kits (Thermo Fisher Scientific, USA) on an ABI 7500 FAST (Thermo Fisher Scientific, USA); 2) tested directly on the Cepheid GeneXpert system using the Xpert Xpress SARS-CoV-2 (Cepheid, USA); or 3) loaded directly into a Roche cobas 6800 system and assayed with the cobas SARS-CoV-2 Test (Roche, Switzerland). The first assay targets the viral S, N, and ORF1ab gene regions. The second targets the viral N and E-gene regions, and the third targets the ORF1ab and E-gene regions. All PCR testing was conducted at the Hamad Medical Corporation Central Laboratory or Sidra Medicine Laboratory, following standardized protocols.

## **Rapid antigen testing**

SARS-CoV-2 antigen tests were performed on nasopharyngeal swabs using one of the following lateral flow antigen tests: Panbio COVID-19 Ag Rapid Test Device (Abbott, USA); SARS-CoV-2 Rapid Antigen Test (Roche, Switzerland); Standard Q COVID-19 Antigen Test (SD Biosensor, Korea); or CareStart COVID-19 Antigen Test (Access Bio, USA). All antigen tests were performed at point-of-care according to each manufacturer's instructions, at public or private hospitals and clinics throughout Qatar, with prior authorization and training by the Ministry of Public Health (MOPH). Antigen test results were electronically reported to the MOPH in real time using the Antigen Test Management System which is integrated with the national COVID-19 database.

# **Section S4. Classification of coexisting conditions.**

The classification of patients and their coexisting conditions was carried out by an independent medical team appointed by the Ministry of Public Health. This process was part of routine needs assessment exercises aimed at planning healthcare use, utilization, and resource allocation.

As Qatar's medical coding systems evolved over time, incorporating different versions of ICD codes as well as SNOMED codes, the medical team developed mappings to harmonize these codes and ensure consistent categorization of coexisting conditions. The study investigators did not have direct access to the detailed procedures, documentation, or specific codes and mappings used by this independent team. Instead, the finalized database of patients and their coexisting conditions was provided to the study investigators exclusively for research purposes.

Coexisting conditions were ascertained and classified based on the mapped codes for the conditions, as recorded in the electronic health record encounters of each individual in the national EMR database that includes all citizens and residents registered in the national and universal public healthcare system. The public healthcare system provides healthcare to the entire resident population of Qatar free of charge or at heavily subsidized costs, including prescription drugs. With the mass expansion of this sector in recent years, facilities have been built to cater to specific needs of subpopulations. For example, tens of facilities have been built, including clinics and hospitals, in localities with high density of craft and manual workers [17].

All encounters for each individual were analyzed to determine the coexisting-condition classification for that individual. The national EMR database includes encounters starting from 2013, when this system was launched in Qatar. Any individual who had at least one encounter with a specific coexisting-condition diagnosis since 2013 was classified as having that coexisting condition. Individuals who do not have records of coexisting-condition encounters in the public healthcare system were classified as having no coexisting conditions.

The classification of coexisting conditions spanned the following conditions: 1) Behchet's disease, 2) cancer, 3) cardiovascular diseases, 4) infectious and parasitic diseases, 5) Crohn's disease, 6) chronic kidney disease (CKD), 7) chronic liver disease (CLD), 8) chronic lung disease, 9) congenital malformations, deformations and chromosomal abnormalities, 10) diseases of the blood and blood-forming organs and certain disorders involving the immune mechanism, 11) diseases of the ear and mastoid process, 12) deep vein thrombosis (DVT), 13) dermatitis, 14) diabetes mellitus, 15) diseases of the circulatory system, 16) diseases of the digestive system, 17) diseases of the eye and adnex, 18) diseases of the genitourinary system, 19) diseases of the musculoskeletal system and connective tissue, 20) diseases of the nervous system, 21) diseases of the respiratory system, 22) diseases of the skin and subcutaneous tissue, 23) endocrine, nutritional and metabolic diseases, 24) gingivitis, 25) hypertension, 26) injury, poisoning and certain other consequences of external causes, 27) mental and behavioral disorders, 28) neoplasms, 29 periodontitis, 30) pregnancy, childbirth and the puerperium, 31) pulmonary tuberculosis, 32) rheumatoid arthritis, 33) Sjogren's syndrome, 34) stroke or neural conditions, 35) symptoms, signs and abnormal clinical and laboratory findings, not elsewhere classified, 36) systemic lupus erythematosus, 37) systemic sclerosis, 38) organ transplant, and 39) other unspecified factors influencing health status and contact with health services.

# **Section S5. Sensitivity analyses.**

**Methods**

***Sensitivity analyses for the impact of temporal and policy-driven changes***

The spectrum of COVID-19 severity evolved over time, shaped by viral evolution and the accumulation of population immunity [12, 18, 19]. Additionally, testing policies experienced shifts, with routine testing becoming less prevalent and symptom-driven testing constituting a larger proportion of conducted tests. These developments may have influenced the study's findings.

To explore this possibility, two sensitivity analyses were performed. The first stratified the study outcomes by the pre- and post-omicron periods, delineated as before and after December 19, 2021 [4]. The second stratified outcomes based on the periods before and after the major changes in testing policies introduced on November 1, 2022 [20]. These analyses were designed to assess the potential impact of these temporal and policy-related changes on the study's conclusions.

***Sensitivity analysis for the impact of misclassified severe, critical, or fatal COVID-19 cases***

The main analysis assumed that all severe, critical, or fatal COVID-19 cases in the population were captured through hospitalization, which is a reasonable assumption given Qatar's healthcare context. It does not seem likely that a severe, critical, or fatal case of COVID-19 would occur without receiving some form of hospital care. To evaluate the impact of this assumption on the study results, a sensitivity analysis was performed under an extreme scenario, assuming that the number of severe, critical, or fatal COVID-19 cases in the population that were not hospitalized was equal to the number of those that were hospitalized.

**Results**

***Sensitivity analyses for the impact of temporal and policy-driven changes***

The cross-tabulation comparing the diagnostic performance of the two hospitalization indicators across the two sensitivity analyses for the impact of temporal and policy-driven changes is presented in Supplementary Table S2, while Table 4 provides the diagnostic performance metrics for these analyses.

The diagnostic performance of any SARS-CoV-2-associated hospitalization, whether in acute-care or ICU beds, as an indicator of severe, critical, or fatal COVID-19 per WHO criteria, was markedly better during the pre-omicron period and prior to the major changes in testing policy than during the post-omicron period and following the policy changes (Table 4).

In contrast, the diagnostic performance of SARS-CoV-2-associated hospitalization exclusively in ICU beds as an indicator of severe, critical, or fatal COVID-19 per WHO criteria demonstrated less variation across these periods. However, it was higher during the pre-omicron period and before the major changes in testing policy compared to the post-omicron period and after the policy changes (Table 4).

***Sensitivity analysis for the impact of misclassified severe, critical, or fatal COVID-19 cases***

The analysis comparing the diagnostic performance of any SARS-CoV-2-associated hospitalization as an indicator of severe, critical, or fatal COVID-19, according to WHO criteria, is presented in Supplementary Table S3. The results demonstrate diagnostic performance metrics consistent with those of the main analysis, including a similar Cohen's kappa value, thereby reaffirming the study findings.

**Table S1. Standards for Reporting Diagnostic Accuracy Studies (STARD) checklist for diagnostic accuracy studies.**

| **Section & Topic** | **No** | **Item** | **Reported on page #** |
| --- | --- | --- | --- |
| **TITLE OR ABSTRACT** | **1** | Identification as a study of diagnostic accuracy using at least one measure of accuracy (such as sensitivity, specificity, predictive values, or AUC) | Abstract |
| **ABSTRACT** | **2** | Structured summary of study design, methods, results, and conclusions (for specific guidance, see STARD for Abstracts) | Abstract |
| **INTRODUCTION** | **3** | Scientific and clinical background, including the intended use and clinical role of the index test | Introduction |
|  | **4** | Study objectives and hypotheses | Introduction |
| **METHODS** | | | |
| *Study design* | **5** | Whether data collection was planned before the index test and reference standard were performed (prospective study) or after (retrospective study) | Methods (‘Acute-care and ICU hospitalizations associated with SARS-CoV-2 infections’, ‘Classification of severe, critical, and fatal COVID-19 according to WHO criteria’, & ‘Study design’) |
| *Participants* | **6** | Eligibility criteria | Methods (‘Study design’) |
|  | **7** | On what basis potentially eligible participants were identified (such as symptoms, results from previous tests, inclusion in registry) | Methods (‘Study design’) |
|  | **8** | Where and when potentially eligible participants were identified (setting, location and dates) | Methods (‘Study population and data sources’ & ‘Study design’) & Section S1 in Supplementary Material |
|  | **9** | Whether participants formed a consecutive, random or convenience series | Not applicable, see Methods (‘Study population and data sources’ & ‘Study design’) & Section S1 in Supplementary Material |
| *Test methods* | **10a** | Index test, in sufficient detail to allow replication | Methods (‘Acute-care and ICU hospitalizations associated with SARS-CoV-2 infections’ & ‘Study design’) |
|  | **10b** | Reference standard, in sufficient detail to allow replication | Methods (‘Classification of severe, critical, and fatal COVID-19 according to WHO criteria’ & ‘Study design’) & Section S2 in Supplementary Material |
|  | **11** | Rationale for choosing the reference standard (if alternatives exist) | Methods (‘Classification of severe, critical, and fatal COVID-19 according to WHO criteria’) |
|  | **12a** | Definition of and rationale for test positivity cut-offs or result categories of the index test, distinguishing pre-specified from exploratory | Methods (‘Acute-care and ICU hospitalizations associated with SARS-CoV-2 infections’ & ‘Study design’) |
|  | **12b** | Definition of and rationale for test positivity cut-offs or result categories of the reference standard, distinguishing pre-specified from exploratory | Methods (‘Classification of severe, critical, and fatal COVID-19 according to WHO criteria’ & ‘Study design’) & Section S2 in Supplementary Material |
|  | **13a** | Whether clinical information and reference standard results were available to the performers/readers of the index test | Methods (‘Classification of severe, critical, and fatal COVID-19 according to WHO criteria’ & ‘Study design’) & Section S2 in Supplementary Material |
|  | **13b** | Whether clinical information and index test results were available to the assessors of the reference standard | Methods (‘Acute-care and ICU hospitalizations associated with SARS-CoV-2 infections’ & ‘Study design’) |
| *Analysis* | **14** | Methods for estimating or comparing measures of diagnostic accuracy | Methods (‘Statistical analysis’) |
|  | **15** | How indeterminate index test or reference standard results were handled | Not applicable, see Methods (‘Acute-care and ICU hospitalizations associated with SARS-CoV-2 infections’, ‘Classification of severe, critical, and fatal COVID-19 according to WHO criteria’, & ‘Study design’) & Section S2 in Supplementary Material |
|  | **16** | How missing data on the index test and reference standard were handled | Not applicable, see Methods (‘Acute-care and ICU hospitalizations associated with SARS-CoV-2 infections’, ‘Classification of severe, critical, and fatal COVID-19 according to WHO criteria’, & ‘Study design’) & Section S2 in Supplementary Material |
|  | **17** | Any analyses of variability in diagnostic accuracy, distinguishing pre-specified from exploratory | Not applicable |
|  | **18** | Intended sample size and how it was determined | Methods (‘Study population and data sources’) & Section S1 in Supplementary Material |
| **RESULTS** | | | |
| *Participants* | **19** | Flow of participants, using a diagram | Results, ‘Figure 1’ |
|  | **20** | Baseline demographic and clinical characteristics of participants | Results (‘Study sample’) & Table 1 |
|  | **21a** | Distribution of severity of disease in those with the target condition | Not applicable |
|  | **21b** | Distribution of alternative diagnoses in those without the target condition | Not applicable |
|  | **22** | Time interval and any clinical interventions between index test and reference standard | Not applicable |
| *Test results* | **23** | Cross tabulation of the index test results (or their distribution) by the results of the reference standard | Results (‘Study sample’), Figure 1, & Table 2, & Table S2 & S3 in Supplementary Material |
|  | **24** | Estimates of diagnostic accuracy and their precision (such as 95% confidence intervals) | Results (‘Any SARS-CoV-2-associated hospitalization as an indicator of infection severity’ & ‘SARS-CoV-2-associated hospitalization in ICU beds as an indicator of infection severity’), Table 3, Table 4, & Table S3 in Supplementary Material |
|  | **25** | Any adverse events from performing the index test or the reference standard | Not applicable, see Methods (‘Acute-care and ICU hospitalizations associated with SARS-CoV-2 infections’, ‘Classification of severe, critical, and fatal COVID-19 according to WHO criteria’) & Section S2 in Supplementary Material |
| **DISCUSSION** | **26** | Study limitations, including sources of potential bias, statistical uncertainty, and generalisability | Discussion, paragraphs 8-14 |
|  | **27** | Implications for practice, including the intended use and clinical role of the index test | Discussion, paragraph 3-7 |
| **OTHER INFORMATION** | **28** | Registration number and name of registry | Not applicable |
|  | **29** | Where the full study protocol can be accessed | Not applicable |
|  | **30** | Sources of funding and other support; role of funders | Acknowledgments & Funding |

# **Table S2. Sensitivity analyses. Study outcomes stratified by pre- and post-omicron periods, as well as before and after major testing policy changes. Cross-tabulation comparing the diagnostic performance of A) any SARS-CoV-2-associated hospitalization in either acute-care or ICU beds as an indicator of severe, critical, or fatal COVID-19 per WHO criteria, and B) SARS-CoV-2-associated hospitalization exclusively in ICU beds as an indicator of severe, critical, or fatal COVID-19 per WHO criteria.**

| **Analyses of pre- and post-omicron periods** | | | | |
| --- | --- | --- | --- | --- |
| **Pre-omicron period^a^** | | | | |
| 1. **Any SARS-CoV-2-associated hospitalization in either acute-care or ICU beds as an indicator of severe, critical, or fatal COVID-19 per WHO criteria** | | | | |
|  | | Confirmed SARS-CoV-2 infections^b^ that progressed to severe, critical, or fatal COVID-19 per WHO criteria | | |
|  |  | Yes | No | Total |
| Confirmed SARS-CoV-2 infections^b^ that were associated with a hospitalization record in either acute-care^c^ or ICU^d^ beds | Yes | 264 | 593 | 857 |
|  | No | 0 | 28,007 | 28,007 |
|  | Total | 264 | 28,600 | 28,864 |
| 1. **SARS-CoV-2-associated hospitalization exclusively in ICU beds as an indicator of severe, critical, or fatal COVID-19 per WHO criteria** | | | | |
|  | | Confirmed SARS-CoV-2 infections^b^ that progressed to severe, critical, or fatal COVID-19 per WHO criteria | | |
|  |  | Yes | No | Total |
| Confirmed SARS-CoV-2 infections^b^ that were associated with a hospitalization record exclusively in ICU beds^d^ | Yes | 127 | 86 | 213 |
|  | No | 137 | 28,514 | 28,651 |
|  | Total | 264 | 28,600 | 28,864 |
| **Omicron period^a^** | | | | |
| 1. **Any SARS-CoV-2-associated hospitalization in either acute-care or ICU beds as an indicator of severe, critical, or fatal COVID-19 per WHO criteria** | | | | |
|  | | Confirmed SARS-CoV-2 infections^b^ that progressed to severe, critical, or fatal COVID-19 per WHO criteria | | |
|  |  | Yes | No | Total |
| Confirmed SARS-CoV-2 infections^b^ that were associated with a hospitalization record in either acute-care^c^ or ICU^d^ beds | Yes | 667 | 8,082 | 8,749 |
|  | No | 0 | 606,563 | 606,563 |
|  | Total | 667 | 614,645 | 615,312 |
| 1. **SARS-CoV-2-associated hospitalization exclusively in ICU beds as an indicator of severe, critical, or fatal COVID-19 per WHO criteria** | | | | |
|  | | Confirmed SARS-CoV-2 infections^b^ that progressed to severe, critical, or fatal COVID-19 per WHO criteria | | |
|  |  | Yes | No | Total |
| Confirmed SARS-CoV-2 infections^b^ that were associated with a hospitalization record exclusively in ICU beds^d^ | Yes | 307 | 387 | 694 |
|  | No | 360 | 614,258 | 614,618 |
|  | Total | 667 | 614,645 | 615,312 |

| **Analyses before and after major testing policy changes** | | | | |
| --- | --- | --- | --- | --- |
| **Before major testing policy changes^e^** | | | | |
| 1. **Any SARS-CoV-2-associated hospitalization in either acute-care or ICU beds as an indicator of severe, critical, or fatal COVID-19 per WHO criteria** | | | | |
|  | | Confirmed SARS-CoV-2 infections^b^ that progressed to severe, critical, or fatal COVID-19 per WHO criteria | | |
|  |  | Yes | No | Total |
| Confirmed SARS-CoV-2 infections^b^ that were associated with a hospitalization record in either acute-care^c^ or ICU^d^ beds | Yes | 789 | 5,846 | 6,635 |
|  | No | 0 | 551,380 | 551,380 |
|  | Total | 789 | 557,226 | 558,015 |
| 1. **SARS-CoV-2-associated hospitalization exclusively in ICU beds as an indicator of severe, critical, or fatal COVID-19 per WHO criteria** | | | | |
|  | | Confirmed SARS-CoV-2 infections^b^ that progressed to severe, critical, or fatal COVID-19 per WHO criteria | | |
|  |  | Yes | No | Total |
| Confirmed SARS-CoV-2 infections^b^ that were associated with a hospitalization record exclusively in ICU beds^d^ | Yes | 386 | 371 | 757 |
|  | No | 403 | 556,855 | 557,258 |
|  | Total | 789 | 557,226 | 558,015 |
| **After major testing policy changes^e^** | | | | |
| 1. **Any SARS-CoV-2-associated hospitalization in either acute-care or ICU beds as an indicator of severe, critical, or fatal COVID-19 per WHO criteria** | | | | |
|  | | Confirmed SARS-CoV-2 infections^b^ that progressed to severe, critical, or fatal COVID-19 per WHO criteria | | |
|  |  | Yes | No | Total |
| Confirmed SARS-CoV-2 infections^b^ that were associated with a hospitalization record in either acute-care^c^ or ICU^d^ beds | Yes | 142 | 2,829 | 2,971 |
|  | No | 0 | 83,190 | 83,190 |
|  | Total | 142 | 86,019 | 86,161 |
| 1. **SARS-CoV-2-associated hospitalization exclusively in ICU beds as an indicator of severe, critical, or fatal COVID-19 per WHO criteria** | | | | |
|  | | Confirmed SARS-CoV-2 infections^b^ that progressed to severe, critical, or fatal COVID-19 per WHO criteria | | |
|  |  | Yes | No | Total |
| Confirmed SARS-CoV-2 infections^b^ that were associated with a hospitalization record exclusively in ICU beds^d^ | Yes | 48 | 102 | 150 |
|  | No | 94 | 85,917 | 86,011 |
|  | Total | 142 | 86,019 | 86,161 |

*Abbreviations*: COVID-19, coronavirus disease 2019; ICU, intensive-care unit; PCR, polymerase chain reaction; RA, rapid antigen; SARS-CoV-2, severe acute respiratory syndrome coronavirus 2; and WHO, World Health Organization.

^a^The omicron period began on December 19, 2021, marking the onset of the first omicron wave.

^b^A confirmed SARS-CoV-2 infection was defined as a documented PCR- or RA-positive test, provided there had not been a previous positive test within the preceding 90 days.

^c^A SARS-CoV-2-associated acute-care hospital admission refers to the admission of an individual with an active SARS-CoV-2 infection to an acute-care bed, irrespective of the individual's clinical condition.

^d^A SARS-CoV-2-associated ICU admission refers to the admission of an individual with an active SARS-CoV-2 infection to an ICU bed, where the ICU-admission clinical team determined that the condition may be related to COVID-19.

^e^The major changes to testing policies were introduced on November 1, 2022.

# **Table S3. Sensitivity analysis. The impact of misclassified severe, critical, or fatal COVID-19 cases. A) Cross-tabulation comparing the diagnostic performance of any SARS-CoV-2-associated hospitalization in either acute-care or ICU beds as an indicator of severe, critical, or fatal COVID-19 per WHO criteria. B) Diagnostic performance metrics for any SARS-CoV-2-associated hospitalization in either acute-care or ICU beds as an indicator of severe, critical, or fatal COVID-19 per WHO criteria.**

| 1. **Cross-tabulation** | | | | |
| --- | --- | --- | --- | --- |
|  | | Confirmed SARS-CoV-2 infections^a^ that progressed to severe, critical, or fatal COVID-19 per WHO criteria | | |
|  |  | Yes | No | Total |
| Confirmed SARS-CoV-2 infections^a^ that were associated with a hospitalization record in either acute-care^b^ or ICU^c^ beds | Yes | 931 | 8,675 | 9,606 |
|  | No | 931 | 633,639 | 634,570 |
|  | Total | 1,862 | 642,314 | 644,176 |
| 1. **Diagnostic performance metrics** | | | | |

| **Diagnostic concordance assessment**  **% (95% CI)** | | **Diagnsotic performance assessment**  **% (95% CI)** | | | |
| --- | --- | --- | --- | --- | --- |
| **Overall percent agreement** | **Cohen's kappa statistic** | **Sensitivity** | **Specificity** | **Positive predicive value** | **Negative predicive value** |
| 98.5  (98.5-98.5) | 0.16  (0.15-0.17) | 50.0  (47.7-52.3) | 98.6  (98.6-98.7) | 9.7  (9.1-10.3) | 99.9  (99.8-99.9) |

*Abbreviations*: COVID-19, coronavirus disease 2019; ICU, intensive-care unit; PCR, polymerase chain reaction; RA, rapid antigen; SARS-CoV-2, severe acute respiratory syndrome coronavirus 2; and WHO, World Health Organization.

^a^A confirmed SARS-CoV-2 infection was defined as a documented PCR- or RA-positive test, provided there had not been a previous positive test within the preceding 90 days.

^b^A SARS-CoV-2-associated acute-care hospital admission refers to the admission of an individual with an active SARS-CoV-2 infection to an acute-care bed, irrespective of the individual's clinical condition.

^c^A SARS-CoV-2-associated ICU admission refers to the admission of an individual with an active SARS-CoV-2 infection to an ICU bed, where the ICU-admission clinical team determined that the condition may be related to COVID-19.

# **References**

1. Altarawneh HN, Chemaitelly H, Ayoub HH, et al. Effects of Previous Infection and Vaccination on Symptomatic Omicron Infections. *N Engl J Med.* Jul 7 2022;387(1):21-34.

2. Abu-Raddad LJ, Chemaitelly H, Ayoub HH, et al. Characterizing the Qatar advanced-phase SARS-CoV-2 epidemic. *Sci Rep.* Mar 18 2021;11(1):6233.

3. Chemaitelly H, Faust JS, Krumholz HM, et al. Short- and longer-term all-cause mortality among SARS-CoV-2- infected individuals and the pull-forward phenomenon in Qatar: a national cohort study. *Int J Infect Dis.* Sep 16 2023;136:81-90.

4. Altarawneh HN, Chemaitelly H, Hasan MR, et al. Protection against the Omicron Variant from Previous SARS-CoV-2 Infection. *N Engl J Med.* Mar 31 2022;386(13):1288-1290.

5. Mahmoud MA, Ayoub HH, Coyle P, et al. SARS-CoV-2 infection and effects of age, sex, comorbidity, and vaccination among older individuals: A national cohort study. *Influenza Other Respir Viruses.* Nov 2023;17(11):e13224.

6. Abu-Raddad LJ, Chemaitelly H, Bertollini R, National Study Group for Covid Vaccination. Effectiveness of mRNA-1273 and BNT162b2 Vaccines in Qatar. *N Engl J Med.* Feb 24 2022;386(8):799-800.

7. Planning and Statistics Authority-State of Qatar. Qatar Monthly Statistics. Available from: <https://www.psa.gov.qa/en/pages/default.aspx>. Accessed on: May 26, 2020. 2020.

8. Chemaitelly H, Bertollini R, Abu-Raddad LJ, National Study Group for Covid Epidemiology. Efficacy of Natural Immunity against SARS-CoV-2 Reinfection with the Beta Variant. *N Engl J Med.* Dec 30 2021;385(27):2585-2586.

9. Chemaitelly H, Ayoub HH, AlMukdad S, et al. Protection from previous natural infection compared with mRNA vaccination against SARS-CoV-2 infection and severe COVID-19 in Qatar: a retrospective cohort study. *Lancet Microbe.* Dec 2022;3(12):e944-e955.

10. Chemaitelly H, Ayoub HH, Tang P, et al. History of primary-series and booster vaccination and protection against Omicron reinfection. *Sci Adv.* Oct 6 2023;9(40):eadh0761.

11. AlNuaimi AA, Chemaitelly H, Semaan S, et al. All-cause and COVID-19 mortality in Qatar during the COVID-19 pandemic. *BMJ Glob Health.* May 2023;8(5).

12. Chemaitelly H, Ayoub HH, Faust JS, et al. Turning point in COVID-19 severity and fatality during the pandemic: a national cohort study in Qatar. *BMJ Public Health.* 2023;1(1):e000479.

13. World Health Organization (WHO). Living guidance for clinical management of COVID-19. Aavailable from: <https://www.who.int/publications/i/item/WHO-2019-nCoV-clinical-2021-2>. Accessed on: February 27, 2023. 2023.

14. World Health Organization (WHO). International Guidelines for Certification and Classification (Coding) of COVID-19 as Cause of Death. Available from: <https://www.who.int/publications/m/item/international-guidelines-for-certification-and-classification-(coding)-of-covid-19-as-cause-of-death>. Accessed on: February 27, 2023. 2023.

15. Abu-Raddad LJ, Chemaitelly H, Ayoub HH, et al. Severity, Criticality, and Fatality of the Severe Acute Respiratory Syndrome Coronavirus 2 (SARS-CoV-2) Beta Variant. *Clin Infect Dis.* Aug 24 2022;75(1):e1188-e1191.

16. Abu-Raddad LJ, Chemaitelly H, Bertollini R, National Study Group for Covid Epidemiology. Severity of SARS-CoV-2 Reinfections as Compared with Primary Infections. *N Engl J Med.* Dec 23 2021;385(26):2487-2489.

17. Al-Thani MH, Farag E, Bertollini R, et al. SARS-CoV-2 Infection Is at Herd Immunity in the Majority Segment of the Population of Qatar. *Open Forum Infect Dis.* Aug 2021;8(8):ofab221.

18. Markov PV, Ghafari M, Beer M, et al. The evolution of SARS-CoV-2. *Nat Rev Microbiol.* Jun 2023;21(6):361-379.

19. Subissi L, von Gottberg A, Thukral L, et al. An early warning system for emerging SARS-CoV-2 variants. *Nat Med.* Jun 2022;28(6):1110-1115.

20. Chemaitelly H, Ayoub HH, AlMukdad S, et al. Bivalent mRNA-1273.214 vaccine effectiveness against SARS-CoV-2 omicron XBB* infections. *J Travel Med.* Sep 5 2023;30(5).
